# Photovoltaic Performance Characterization of Textured Silicon Solar Cells Using Luminescent Down-Shifting Eu-Doped Phosphor Particles of Various Dimensions

**DOI:** 10.3390/ma10010021

**Published:** 2017-01-01

**Authors:** Wen-Jeng Ho, Yu-Jie Deng, Jheng-Jie Liu, Sheng-Kai Feng, Jian-Cheng Lin

**Affiliations:** Department of Electro-Optical Engineering, National Taipei University of Technology, No. 1, Section 3, Zhongxial East Road, Taipei 10608, Taiwan; t103658035@ntut.edu.tw (Y.-J.D.); jjliu@ntut.edu.tw (J.-J.L.); ntw1000@gmail.com (S.-K.F.); t104658023@ntut.edu.tw (J.-C.L.)

**Keywords:** Eu-doped phosphor, luminescent downshifting (LDS), textured silicon solar cells

## Abstract

This paper reports on efforts to enhance the photovoltaic performance of textured silicon solar cells through the application of a layer of Eu-doped silicate phosphor with particles of various dimensions using the spin-on film technique. We examined the surface profile and dimensions of the Eu-doped phosphors in the silicate layer using optical microscopy with J-image software. Optical reflectance, photoluminescence, and external quantum efficiency were used to characterize the luminescent downshifting (LDS) and light scattering of the Eu-doped silicate phosphor layer. Current density-voltage curves under AM 1.5G simulation were used to confirm the contribution of LDS and light scattering produced by phosphor particles of various dimensions. Experiment results reveal that smaller phosphor particles have a more pronounced effect on LDS and a slight shading of incident light. The application of small Eu-doped phosphor particles increased the conversion efficiency by 9.2% (from 12.56% to 13.86%), far exceeding the 5.6% improvement (from 12.54% to 13.32%) achieved by applying a 250 nm layer of SiO_2_ and the 4.5% improvement (from 12.37% to 12.98%) observed in cells with large Eu-doped phosphor particles.

## 1. Introduction

The conversion efficiency of solar cells is limited by optical absorption, carrier transport, and carrier collection. The maximum theoretical efficiency of a single-junction crystalline-silicon (C-Si) solar cell is 31% under AM 1.5G illumination [1]. This limitation can be attributed to losses associated with the excess energy of above-bandgap photons, photon transparency below the band gap, and radiative and Auger recombination. The efficiency of solar cells is also affected by the surface reflection of incident solar radiation and/or the unproductive absorption of light at the back contact, losses due to the recombination of photo-generated carriers, losses due to recombination, and losses at contacts. A number of methods have been devised to reduce these losses. Researchers have achieved efficiency of 25% in the laboratory using passivated emitter and rear locally diffused (PERL) solar cells [2]; however, the ultimate challenge is to engineer solutions that could be produced in high volumes at low cost. Nanomaterials are currently being employed in photovoltaics to reduce the fundamental spectral losses in single-junction silicon solar cells, which can reach 50% [3]. Modification of the spectrum using down- and/or up-conversion or shifting is a relatively straightforward and cost-effective means of enhancing the conversion efficiency of single-junction cells [4,5,6,7,8,9,10,11,12,13,14,15]. The conversion efficiency of C-Si solar cells is relatively low, due to high reflectance and low spectral response at ultraviolet (UV) and blue wavelengths (300–450 nm). Incident photons of higher energy (within UV-blue wavelengths) are absorbed within a short distance from the surface, which results in high recombination loss. Many researchers have sought to enhance the overall conversion efficiency by applying a down-conversion (DC) layer or a down-shifting (DS) layer over the top surface of the C-Si in order to improve its spectral response in the UV-blue region [16,17,18]. Other researchers have developed phosphor materials for luminescent downshifting (LDS) with the aim of converting high-energy incident photons into lower energy photons in photovoltaic devices [19,20,21,22,23,24,25].

This study characterized the coverage, LDS, and reflectance of a layer of Europium-doped (Eu-doped) silicate phosphor particles of various dimensions containing 3 wt % Eu-doped silicate phosphors, for use in coating textured-type C-Si solar cells. Optical reflectance, photoluminescence (PL), and external quantum efficiency (EQE) measurements were used to characterize the LDS and optical properties of the Eu-doped silicate phosphor layer. We also investigated the LDS, light scattering, and light shading properties of a Eu-doped silicate phosphor layer deposited on bare textured C-Si solar cells and the influence of phosphor particle dimensions. EQE response and photovoltaic current density-voltage (J-V) characteristics under AM 1.5G simulation measurements were used to quantify improvements in photovoltaic performance as a function of LDS and particle dimensions.

## 2. Experiment

### 2.1. Characterization of Eu-Doped Silicate Phosphor Layer

This study employed Eu-doped silicate phosphors ((Sr_1−x_Ba_x_)_2_SiO_4_:Eu^2+^F; O6040TM; InteMatix Company, Fremont, CA, USA) with dimensions of 15–25 μm (referred hereafter as large particles) to induce LDS. Phosphor particles of 5–15 μm (referred to as medium particles) and 2–5 μm (referred to as small particles) were obtained by grinding larger silicate phosphor particles in an agate mortar for approximately 30–60 min. Powders of 0.06 g Eu-doped silicate phosphor particles (large, medium, and small phosphor particles) were respectively mixed with a 1.94 g silica film solution (Emulsitone Company, Whippany, NJ, USA). Planar C-Si substrates were coated with these solutions using the spin-on film technique at 3000 rpm for 60 s, before being baked at 200 °C for 30 min under an air atmosphere. This resulted in a layer that included 3 wt % Eu-doped silicate phosphors particles on the planar C-Si substrates. For comparison, we also produced a planar C-Si substrates, which was coated with a SiO_2_ layer (using the same Silicafilm solution), i.e., without any phosphor particles. The surface morphology of the Eu-doped silicate phosphor layer was examined using SEM (Hitachi S-4700, Hitachi High-Tech Fielding Corporation, Tokyo, Japan). The LDS and light scattering and shading properties of Eu-doped silicate phosphor layer were characterized according to PL spectra (Ramboss 500i Micro-PL Spectroscopy, Spectrolab, Newbury, UK) and optical reflectance using an UV/VIS/NIR spectrophotometer (Lambda 35, PerkinElmer Inc., Waltham, MA, USA).

### 2.2. Fabrication and Characterization of Textured C-Si Solar Cells Coated with a Eu-Doped Phosphor Layer

**Figure 1:**
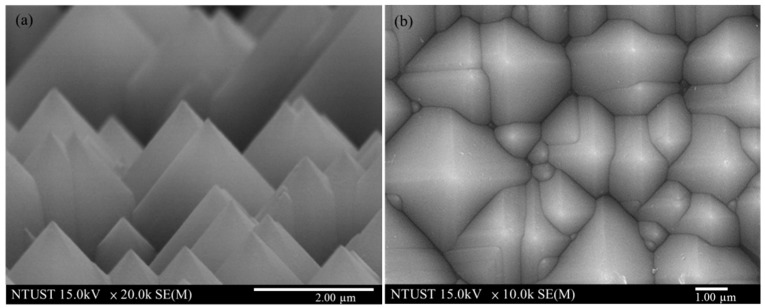
SEM images of textured surface etched using an anisotropic solution of H_2_O/KOH/IPA at 80 °C for 20 min in (**a**) side-view; (**b**) top-view.

**Figure 2:**
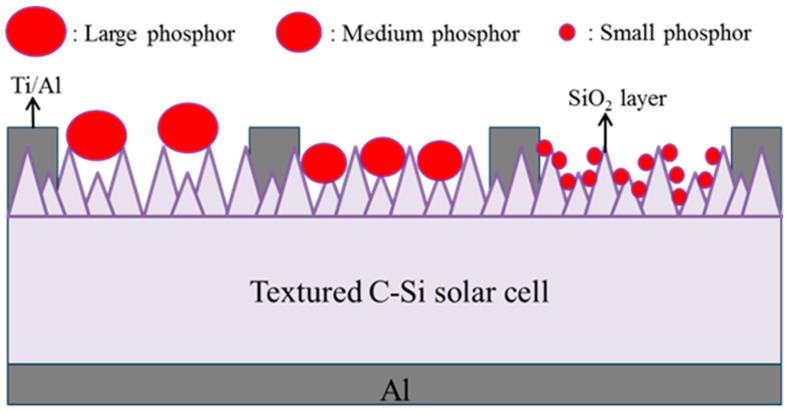
Schematic diagram of textured C-Si solar cell coated with a layer of Eu-doped silicate phosphor particles.

Czochralski (CZ)-grown boron-doped C-Si wafers (525 μm-thick) with a resistivity of a 10 Ω·cm (100) orientation were first cleaned using a standard RCA (Radio Corporation of America) cleaning process. The surface of the C-Si substrate was then etched by being dipped in an anisotropic solution of H_2_O/KOH/IPA at 80 °C for 20 min to produce a pyramidal surface structure. Top-view and side-view SEM images of the randomly etched surface are presented in Figure 1a,b. The minimum and maximum spacing between pyramids were respectively 3 μm and 7 μm, whereas the minimum and maximum heights were 3 μm and 7 μm. An n^+^-Si emitter layer (0.3 μm-thick) with a sheet resistance of approximately 80 Ω/sq was applied to the textured C-Si substrates using a POCl_3_ diffusion process in a tube diffusion chamber at 850 °C over a period of 3 min. Any phosphorous silicate glass remaining on the surface was removed using a buffered oxide etchant prior to deposition of the electrode films. Aluminum (Al) film with a thickness of 500 nm was deposited on the rear surface using electron-beam (E-beam) evaporation and annealed in an RTA chamber at 450 °C for 5 min under ambient N_2_/H_2_ to form a back electrode. Finally, top contact grid-electrodes comprising a 20 nm titanium (Ti) film and a 300 nm Al film were fabricated using lift-off photolithography and E-beam evaporation to produce bare textured C-Si solar cells. Silicate solutions containing 3 wt % Eu-doped silicate phosphors were deposited on the bare textured C-Si solar cells using the spin-on film deposition. Figure 2 presents a schematic diagram of a textured C-Si solar cell coated with a layer of Eu-doped silicate phosphor particles. For comparison, we also produced a textured Si solar cell that was coated with a layer of SiO_2_ (using the same Silicafilm solution), i.e., without any phosphor particles. The resulting rough surfaces produced by the phosphor particles were examined using SEM. The optical reflectance of all solar cells was characterized using an UV/VIS/NIR spectrophotometer. The external quantum efficiency (EQE) of the cells was also measured over a range of wavelengths from 350 to 1100 nm using a solar cell spectral response measurement system (EQE-RQE-R3015, Enli Technology Co., Ltd., Kaohsiung, Taiwan). The photovoltaic current-voltage (I-V) characteristics of the proposed cells were measured using a solar simulator (XES-151S, San-Ei Electric Co., Ltd., Osaka, Japan) and source meter (Keithley 2400, Keithley Instruments, Inc., Cleveland, OH, USA) at 25 °C. The solar simulator was calibrated according to an NREL-certified crystalline silicon reference cell (PVM-894, PV Measurements Inc., Boulder, CO, USA) before obtaining measurements.

## 3. Results and Discussion

**Figure 3:**
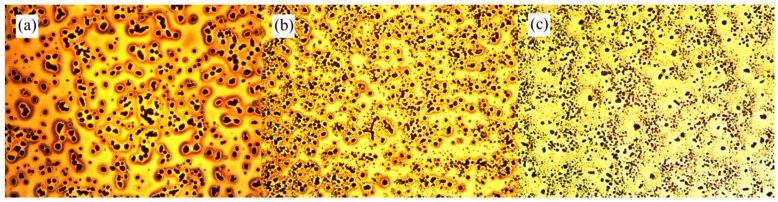
Optical microscope (OM) images (multiplicative: ×500) of planar C-Si substrates coated with a layer of Eu-doped silicate phosphors: (**a**) large; (**b**) medium; (**c**) small particles.

Figure 3 presents top-view optical microscope (OM) images (multiplicative: ×500) of planar C-Si substrates coated with an Eu-doped silicate phosphor layer comprising (a) large; (b) medium; and (c) small particles. The coverage and average dimensions are as follows: large particles (12.35% and 16.47 μm), medium particles (16.18% and 8.49 μm), small particles (21.35% and 3.66 μm), as calculated from OM images using Image J software. These results indicate that some of the particles exceeded 30 μm, due to the aggregation of some of the phosphor particles. Denser coverage could be expected to produce stronger LDS effects, and larger particles could be expected to increase light shading. We therefore sought to distribute a layer of Eu-doped silicate phosphors of appropriate dimensions at an appropriate density uniformly across the textured surface.

**Figure 4:**
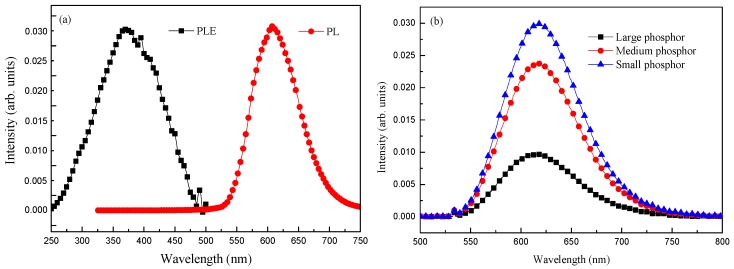
(**a**) PL excitation and PL fluorescence from a layer comprising 3 wt % Eu-doped silicate phosphor deposited on a C-Si substrate; (**b**) PL intensity as a function of phosphor particles coverage.

Figure 4a presents the PL excitation (PLE; i.e., absorption) and PL fluorescence (radiation or emission) of a layer comprising 3 wt % Eu-doped silicate phosphors deposited on a C-Si substrate. The peak PLE of Eu-doped silicate phosphor was measured at approximately 375 nm with a full width at half maximum (FWHM) of approximately 110 nm, which means that incident photons of higher energy (within wavelengths of 260–480 nm) were absorbed by the Eu-doped silicate phosphor. In contrast, peak PL emissions at approximately 610 nm with FWHM of approximately 100 nm indicates the absorption of high-energy photon, resulting in the emission of visible photons at wavelengths of 525–725 nm. When a phosphor-material system absorbs a photon, it generally gains energy and enters an excited state. One way for the phosphor-material system to relax is through the emission of a photon and its associated energy. When the energy of the emitted photon is less than that of the absorbed photon, the difference in energy is referred to as the Stokes shift. In this study, the Stoke shift of the Eu-doped silicate phosphor was approximately 235 nm. The measured PL emission and PLE absorption results demonstrate that the 3 wt % Eu-doped silicate phosphor layer absorbed UV photons and converted them into visible photons, as indicated by the excellent LDS behavior. Figure 4b presents PL intensity as a function of phosphor particle coverage. We obtained higher PL intensities from samples with denser particle coverage. Under the same 3 wt % condition, the number of small particles far exceeds that of large particles, thereby providing far more Eu-doped phosphors to absorb incident photons of higher energy. The measured PL emission results revealed that 3 wt % Eu-doped silicate phosphor layers with denser coverage of smaller particles achieved impressive LDS performance thanks to high PL intensity.

**Figure 5:**
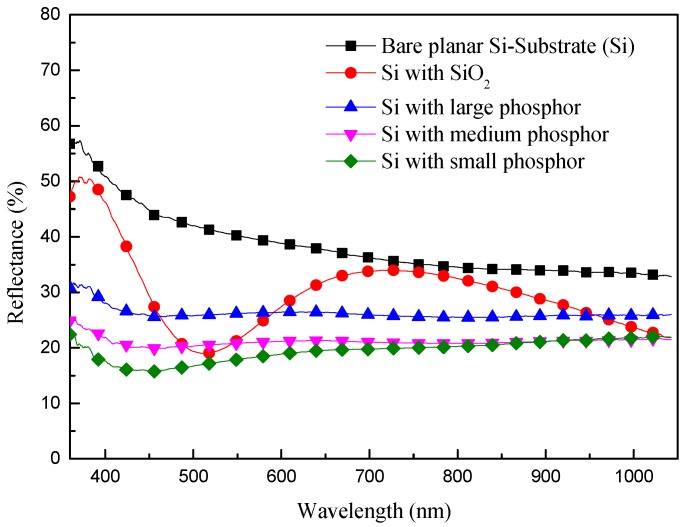
Optical reflectance of a bare planar Si substrate, a Si substrate with SiO_2_ layer, and Si substrates with large, medium, and small phosphor particles disseminated within the SiO_2_ layer.

Figure 5 illustrates the optical reflectance of planar C-Si substrates with the following configurations: a bare Si substrate, a Si substrate with a SiO_2_ layer of 250 nm, and a Si substrate with either large, medium, or small phosphor particles disseminated within a SiO_2_ layer. The reflectance of the Si substrate was altered by the application of a SiO_2_ layer, and the lowest reflectance value was observed at 525 nm, due to the effects of destructive interference. Compared with the bare Si substrate, the sample with the SiO_2_ layer presented typical anti-refractive properties. In contrast, the sample with Eu-doped silicate phosphors presented a reduction in reflectance across the entire range of wavelengths. The reflectance of the sample with Eu-doped silicate phosphors was lower than that with only a SiO_2_ layer at wavelengths below 450 nm, due to the absorption of high-energy photons by the phosphor particles. Furthermore, samples with denser coverage presented higher absorption values. Within a wavelength range of 730–1200 nm, the decrease in reflectance can be attributed to forward light scattering by the phosphor particles. The higher reflectance of samples with large phosphor particles (at 525 nm) can be attributed to a reduction in destructive interference and the slightly higher reflective properties of larger particles. Therefore, the optical reflectance results examine clearly the LDS, reflection, and light scattering effects on Si substrate coated with various phosphor particles mixed in a SiO_2_ layer, compared with the Si substrate coated with a SiO_2_ layer. Furthermore, the smaller particles (denser coverage) produced stronger LDS and light scattering effects. We used the LDS, reflectivity, and light scattering results from planar C-Si substrates coated with phosphor particles as a baseline by which to evaluate the performance of textured C-Si solar cells in various configurations.

**Figure 6:**
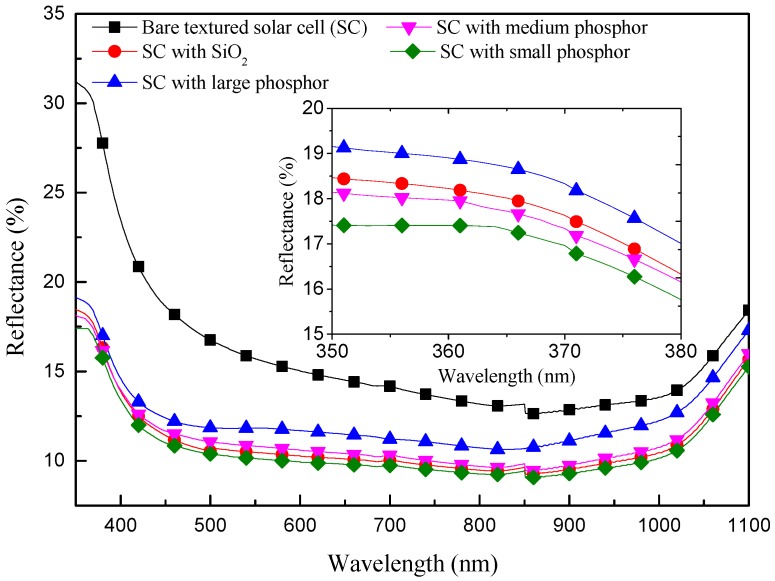
Optical reflectance of C-Si solar cells with the following configurations: a bare textured cell, a textured cell with a SiO_2_ layer, and textured cells with Eu-doped silicate phosphor (large, medium, and small particles).

Figure 6 presents the optical reflectance of C-Si solar cells with the following configurations: a bare textured cell, a textured cell with a 250 nm layer of SiO_2_, and textured cells coated with Eu-doped silicate phosphor particles (large, medium, and small) applied over a SiO_2_ layer. The reflectance of the textured cell was reduced by the application of either a SiO_2_ layer or a layer of Eu-doped silicate phosphor particles. Across the entire range of wavelengths, the reflectance of the textured cell with large phosphor particles was higher than that of the textured cell with a SiO_2_ layer. The large phosphor particles situated near the top of the pyramids (Figure 2) interfered with the anti-reflective properties of the texturing. The surface of the large particles reflected some of the incident light and shaded the pyramidal structures beneath, thereby altering their anti-reflection and multi-reflective functionality. In contrast, the reflectance of the cell with small phosphor particles was monolithically reduced across the entire range of wavelengths, compared to the textured cell with only a SiO_2_ layer. In this work, the diameter of the small phosphor particles was less than that the spacing between pyramids (Figure 2). Thus, the small particles did not undermine the anti-reflective or multiple-reflective benefits of the pyramidal structures, and the incident light was forward scattered into the cell by the small phosphor particles, resulting in lower optical reflectance from 350 to 1100 nm. Furthermore, the sample with small phosphor particles presented the lowest reflectance at 350–370 nm due to the absorption of high-energy incident photons by the small Eu-doped phosphor particles.

**Figure 7:**
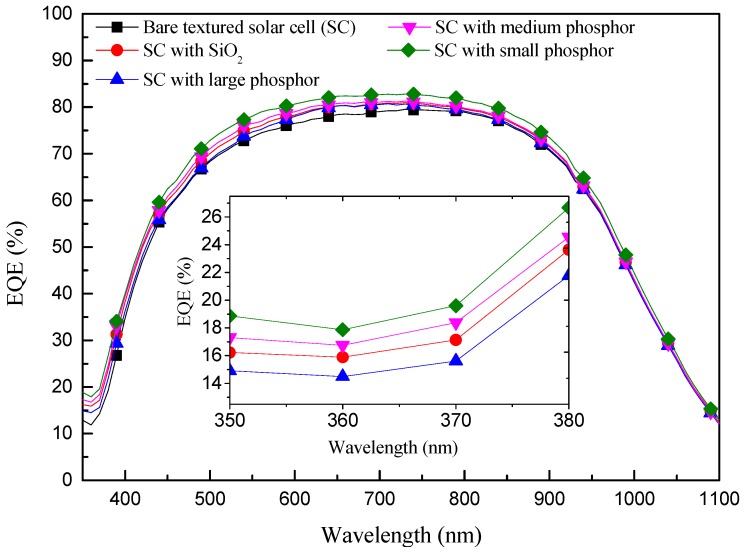
EQE response of C-Si solar cells with the following configurations: a bare textured cell, a textured cell with SiO_2_ layer, and textured cells with Eu-doped silicate phosphor particles (large, medium, and small).

Figure 7 presents the EQE response of C-Si solar cells with the following configurations: a bare textured cell, a textured cell with a SiO_2_ layer of 250 nm, and textured cells with Eu-doped phosphor particles applied over a SiO_2_ layer (large, medium, and small). The EQE values of solar cells coated with Eu-doped phosphor particles were higher at wavelengths of 350–370 nm due to the effects of LDS. Furthermore, the EQE response of the cell with small phosphor particles was superior to that of cells with medium and large phosphor particles. The LDS exhibited by the cell with small phosphor particles was due to the large number of the particles (high surface coverage), far exceeding that of the cells with medium or large particles. The cell with small phosphor particles also exhibited a more pronounced increase in EQE via forward light scattering. These findings are in good agreement with those of reflectance. We also compared the average weighted EQE (*EQE_W_*) of a bare textured cell, a textured cell with a SiO_2_ layer of 250 nm, and textured cells with Eu-doped phosphor particles (large, medium, and small) at wavelengths (λ) from 350 to 1100 nm. *EQE_W_* was calculated using Equation (1).

(1) $$EQE_{W}=\frac{\int_{\lambda_{\min}}^{\lambda_{\max}}EQE\left(\lambda \right)\times \phi_{ph}\left(\lambda \right)}{\int_{\lambda_{\min}}^{\lambda_{\max}}\phi_{ph}\left(\lambda \right)}$$

where *φ_ph_*(λ) is the photon flux of AM 1.5G solar energy spectrum as a function of wavelength. The resulting *EQE_W_* values are listed in Table 1. These results demonstrate that small Eu-doped phosphor particles on a textured cell can enhance EQE at UV-wavelengths via LDS and at long-wavelengths via light scattering.

**Figure 8:**
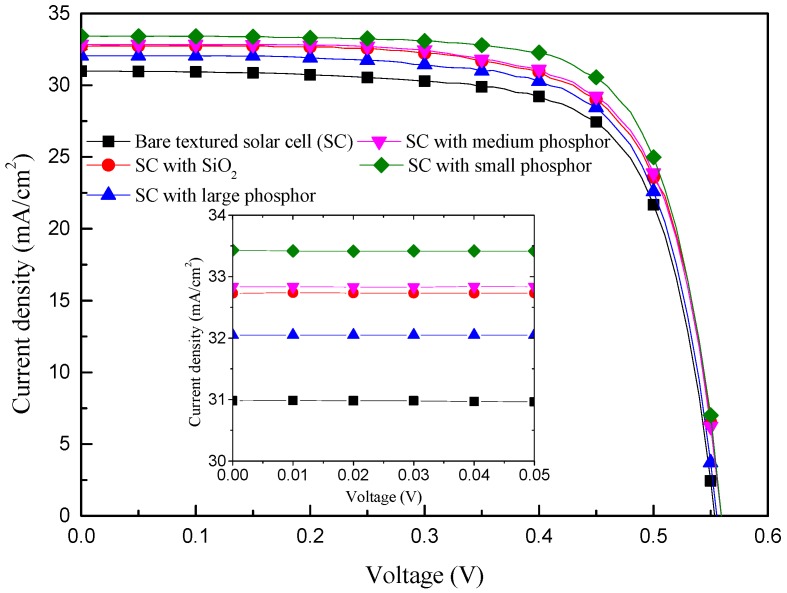
Photovoltaic current density-voltage curves of C-Si solar cells with the following configurations: a bare textured cell, a textured cell with SiO_2_ layer of 250 nm, and textured cells with Eu-doped phosphor particles (large, medium, and small) applied over a layer of SiO_2_.

Figure 8 presents the photovoltaic current density-voltage curves of C-Si solar cells with the following configurations: a bare textured cell, a textured cell with a SiO_2_ layer of 250 nm, and textured cells with Eu-doped phosphor particles (large, medium, and small) applied over a SiO_2_ layer. The photovoltaic performance of all evaluated solar cells is summarized in Table 2. We averaged the short-circuit current density (J_sc_), open-circuit voltage (V_oc_), and conversion efficiency (η) of the four bare-textured cells used in this study as 30.76 mA/cm^2^, 558.37 mV, and 12.475%. These values were used in subsequent comparisons. The application of a 250 nm layer of SiO_2_ increased the J_sc_ of the cells by 5.6% (from 30.98 to 32.73 mA/cm^2^) due to the anti-reflective properties of the SiO_2_. The application of Eu-doped phosphor particles over a SiO_2_ layer led to the following improvements in J_sc_: for small particles, a 9.2% increase (from 30.62 to 33.43 mA/cm^2^); for medium particles, a 6.8% increase (from 30.75 to 32.83 mA/cm^2^); for large particles, a 4.5% increase (from 30.67 to 32.05 mA/cm^2^). These improvements can be attributed to the combined contributions of anti-reflectivity and LDS. Note that the J_sc_ of these solar cells was strongly correlated with the intensity of the PL signal, EQE response, and EQEW values. The small Eu-doped phosphor particles had a greater effect on J_sc_ than did the large particles or the SiO_2_ layer due to their high EQE response. Solar cell efficiency generally depends on J_sc_, V_oc_, and the fill factor (FF); however, we observed variations of less than 2% in V_oc_ and FF among the cells with different surface structure profiles. The factors that made the greatest contribution to η were J_sc_ and EQE. The application of small Eu-doped phosphor particles on a textured C-Si solar cell led to a 1.30% increase in absolute conversion efficiency (from 12.56% to 13.86%) compared to that of a 0.78% (from 12.54% to 13.32%) for a bare textured cell with a SiO_2_ layer of 250 nm.

## 4. Conclusions

This paper reports on the fabrication and characterization of textured C-Si solar cells coated with a layer of LDS Eu-doped silicate phosphor particles of various dimensions. The electrical and optical properties were shown to depend largely on the dimensions of phosphor particles. The application of smaller Eu-doped phosphor particles to textured solar cells led to significant improvements in external quantum efficiency and short-circuits current density. The factors that made the greatest contribution to η were J_sc_ and EQE. The application of small Eu-doped phosphor particles on a textured C-Si solar cell led to a 1.30% increase in absolute conversion efficiency (from 12.56% to 13.86%) compared to that of a 0.78% (from 12.54% to 13.32%) for a bare textured cell with a SiO_2_ layer of 250 nm.

## Figures and Tables

**Table 1:** Average weighted EQE (*EQE_W_*) of all evaluated solar cells.

Cell Type	*EQE_W_* (%)
Bare Textured Cell (SC)	67.31
SC with a SiO_2_ Layer	68.83
SC with Large Phosphor Particles	68.53
SC with Medium Phosphor Particles	69.41
SC with Small Phosphor Particles	70.91

**Table 2:** Photovoltaic performance of all evaluated solar cells.

Cell Type	J_sc_ (mA/cm^2^)	V_oc_ (mV)	FF (%)	η (%)
Bare Textured Cell (SC)	30.98	554.93	72.94	12.54
SC with a SiO_2_ Layer	32.73	559.40	72.75	13.32
Bare Textured Cell (SC)	30.67	554.51	72.74	12.37
SC with Large Phosphor Particles	32.05	556.67	72.70	12.98
Bare Textured Cell (SC)	30.75	556.98	72.51	12.43
Cell with Medium Phosphor Particles	32.83	559.34	72.75	13.36
Bare Textured Cell (SC)	30.62	557.07	73.61	12.56
SC with Small Phosphor Particles	33.43	560.77	73.94	13.86
